# Downregulation of ZC3H13 by miR-362-3p/miR-425-5p is associated with a poor prognosis and adverse outcomes in hepatocellular carcinoma

**DOI:** 10.18632/aging.203939

**Published:** 2022-03-12

**Authors:** Shuang Wu, Shihai Liu, Yongxian Cao, Geng Chao, Peng Wang, Huazheng Pan

**Affiliations:** 1 Department of Clinical Laboratory, The Affiliated Hospital of Qingdao University, Qingdao 266003, Shandong, People’s Republic of China; 2 Department of Medicine, Qingdao University, Qingdao 266071, Shandong, People’s Republic of China; 3 Medical Animal Laboratory, The Affiliated Hospital of Qingdao University, Qingdao 266003, Shandong, People’s Republic of China; 4 Department of Oncology, Weifang Yidu Central Hospital, Qingzhou 262509, Shandong, People’s Republic of China

**Keywords:** miRNA, prognosis, HCC, m6A

## Abstract

Hepatocellular carcinoma (HCC) is notorious for its poor prognosis. Previous studies identified several N6-methyladenosine (m6A)-related genes that play key roles in the initiation and progression of HCC patients. In particular, the N6-methyladenosine RNA methylation regulator ZC3H13 could be a candidate as a novel biomarker and therapeutic target for hepatocellular carcinoma. In HCC, low expression of ZC3H13 was reported, but the molecular reason is unclear. In this study, we performed pan cancer analysis for ZC3H13 expression and prognosis using The Cancer Genome Atlas (TCGA) and Genotype-Tissue Expression (GTEx) data and found that ZC3H13 might be a potential tumor suppressor gene in HCC. Subsequently, miRNAs contributing to ZC3H13 downregulation were identified by a series of *in silico* analyses, including expression analysis, correlation analysis, and survival analysis. Finally, the miR-362-3p/miR-425-5p-ZC3H13 axis was identified as the most likely upstream miRNA-related pathway of ZC3H13 in HCC. Additionally, miR-362-3p/miR-425-5p mimic and inhibitor results were detected by quantitative real-time PCR (qPCR) analysis and western blotting. We identified an upstream regulatory mechanism of ZC3H13 in HCC, namely, the miR-362-3p/miR-425-5p-ZC3H13 axis. Moreover, the ZC3H13 level was significantly positively associated with tumor immune cell infiltration, biomarkers of immune cells, and immune checkpoint expression. Collectively, our findings elucidated that ncRNA-mediated downregulation of ZC3H13 was correlated with a poor prognosis and tumor immune infiltration in HCC. In conclusion, this study demonstrates that ZC3H13 is a direct target of miR-362-3p/miR-425-5p in liver hepatocellular carcinoma (LIHC) that regulates immune modulation in the microenvironment of LIHC.

## INTRODUCTION

Hepatocellular carcinoma (HCC) is the third most common cause of cancer death worldwide [1]. To date, chronic hepatitis B and C virus (HBV/HCV) infection, obesity, type 2 diabetes, alcohol abuse, smoking and aflatoxins have been clearly identified as risk factors for the development of liver cancer [2]. The difficulty in diagnosing hepatocellular carcinoma at an early stage is due to the lack of typical clinical symptoms in patients with HCC. Once diagnosed, surgical resection is the most effective treatment [3]. Currently, with the advancement of medicine, considerable progress has been made in the diagnosis, treatment and prognosis, but the prognosis of HCC patients remains unsatisfactory, and more than 700,000 people still die each year [4]. Because of this high lethality rate and high risk of recurrence in postoperative patients, dynamic surveillance of hepatocellular carcinoma is necessary [5]. Therefore, identifying promising HCC prognostic biomarkers and developing effective therapeutic targets are the main tasks of current studies.

Tumorigenesis and progression are driven by a combination of genetic, epigenetic, and environmental factors. Epigenetic factors can be vividly likened to a bridge to describe the critical role of genetic and environmental factors [6]. N6-methyladenosine (m6A) was known for being the most important modification of mRNA and for its importance in post-transcriptional epigenetic modifications [7, 8].

Regulation of m6A methyltransferase (“writers”), m6A demethylase (“erasers”) and m6A read-binding protein (“readers”) together participate in M6A modification, forming a dynamic and reversible process [9]. Methyltransferases are responsible for adding a methyl group to the nitrogen on the sixth carbon of the aromatic ring of adenosine residues, hence the name “writers”. Factors with these roles include methyltransferase 14 (METTL14), VIRMA (formerly KIAA1429), putative RNA binding protein 15 (RBM15), Wilms tumor 1 associated protein (WTAP), and zinc finger CCCH type 13 containing (ZC3H13) [10].

ZC3H13, one of the “writers”, is involved in cancers. For example, Gong et al. demonstrated that ZC3H13 can be used as a negative regulator to assess the prognosis of breast cancer patients. The downregulation of ZC3H13 expression in breast cancer patients coincides with the fact that this downregulation promotes breast cancer invasion [11]. A recent report by Zhu et al. found that overexpression of ZC3H13 could impair the proliferation and invasion of colorectal cancer cells. It is possible that the mechanism involved is to act as an upstream regulator involved in the Ras-ERK signaling pathway to achieve the above inhibitory effects [12]. The current study suggests that ZC3H13 may act as an upstream regulator of the Ras-ERK signaling pathway to achieve inhibition of CRC invasion and proliferation.

Considering the widespread concern of the methyltransferase ZC3H13, researchers have proposed the following views: ZC3H13 is expected to be a marker for evaluating the prognosis of patients with hepatocellular carcinoma [13]. However, the expression of ZC3H13 in patients with hepatocellular carcinoma remains controversial, which triggered us to delve into the expression level of ZC3H13 in patients with hepatocellular carcinoma and its regulatory factors.

To resolve the current controversy concerning the expression level of ZC3H13 in hepatocellular carcinoma, we further clarified whether ZC3H13 could be a prognostic marker to evaluate hepatocellular carcinoma patients. The present study is based on the TCGA dataset of the prognostic significance of low ZC3H13 levels in hepatocellular carcinoma. More meaningfully, we further explained the reason for the low expression of ZC3H13 at the miRNA level through bioinformatics and experimental verification, which provided a reliable theoretical basis for this study.

## RESULTS

### Pan cancer analysis of ZC3H13

**Figure 1:**
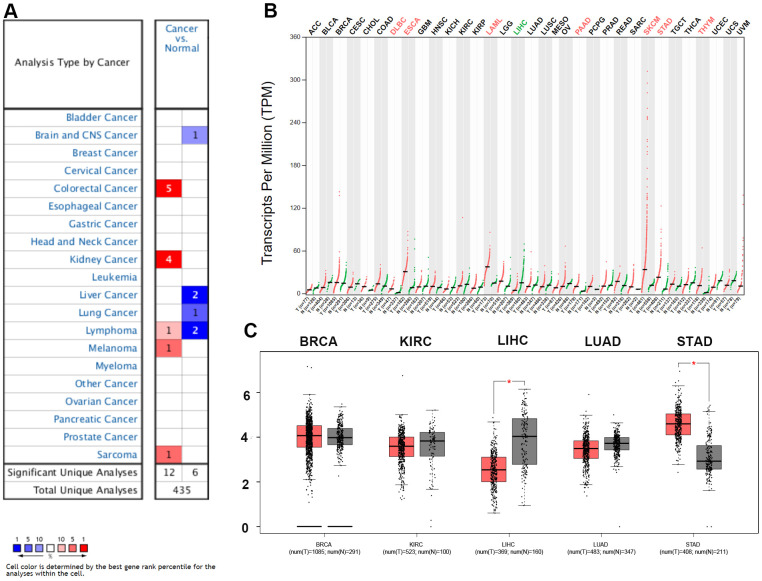
**Expression analysis for ZC3H13 in multiple cancers.** (**A**) mRNA expression levels of ZC3H13 in various types of cancer (Oncomine). The threshold was designed with the following parameters: fold change = 2 and P value = 0.01. The cell number represents the number of datasets that meets the threshold. The color intensity (RED OR BLUE) is directly proportional to the significance level of upregulation or downregulation. (**B**, **C**) ZC3H13 expression in TCGA (BRCA, KIRC, LIHC, LUAD, STAD) tissues compared with the corresponding TCGA and GTEx normal tissues. *p value < 0.05.

In this study, the expression of ZC3H13 in different tumors was initially assessed by the Oncomine database. Among them, ZC3H13 was highly expressed in five cancers, including colorectal cancer, kidney cancer, lymphoma, melanoma and sarcoma. But downregulated in four cancers, brain and CNS cancer, liver cancer, lung cancer, and lymphoma (Figure 1A). Next, to make our analysis more reliable, the expression of ZC3H13 in different cancers was further validated by the GEPIA database. The results showed that ZC3H13 expression was significantly increased in patients with DLBC, ESCA, LAML, PAAD, SKCM, STAD and THYM (Figure 1B). In LIHC, ZC3H13 was significantly decreased (Figure 1C). Taken together, both database results confirmed that ZC3H13 is downregulated in HCC, suggesting that abnormal ZC3H13 may drive the carcinogenesis of HCC.

### ZC3H13 can evaluate the prognosis of HCC patients

**Figure 2:**
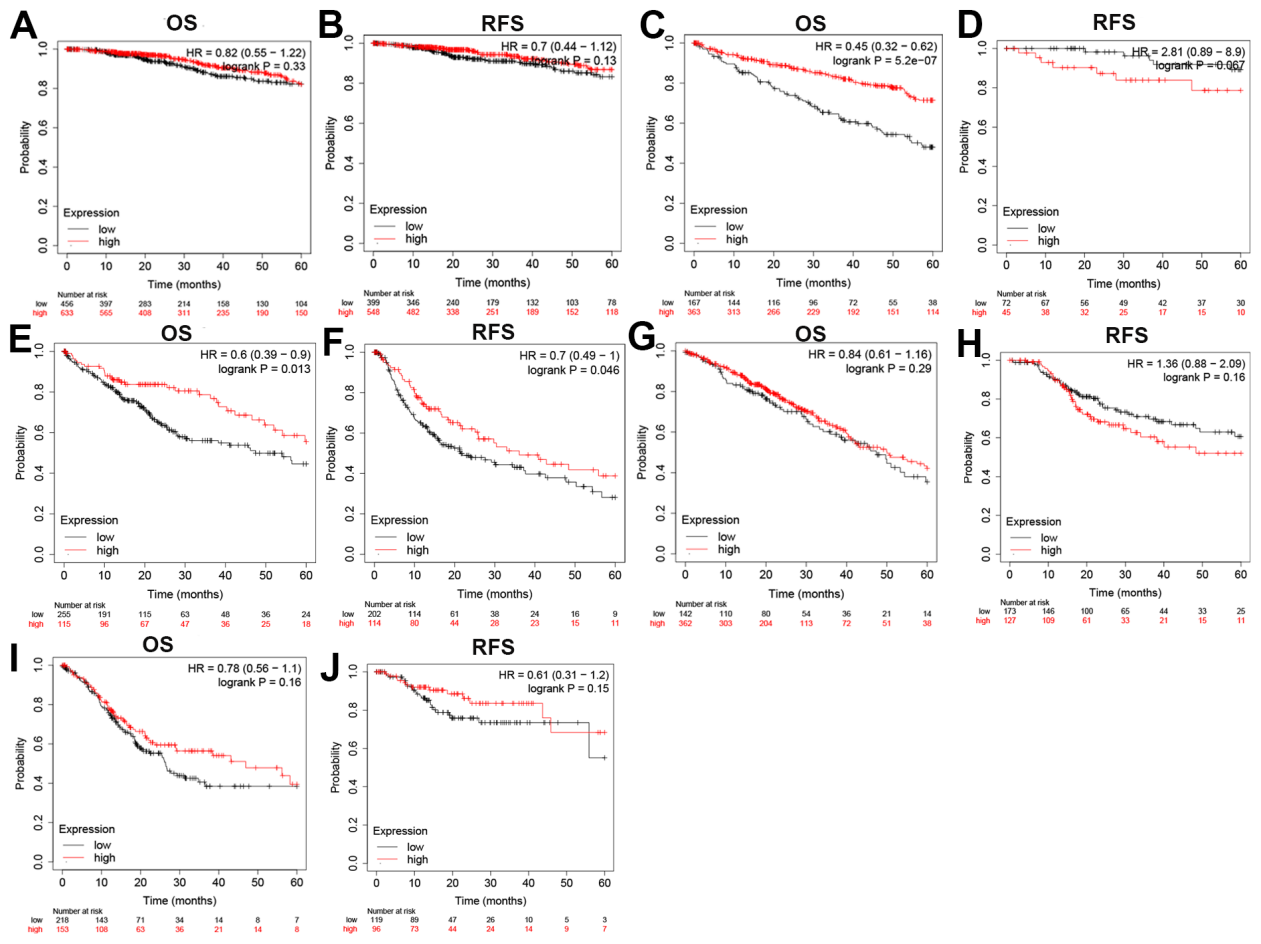
**Overall survival (OS) and disease-free survival (RFS) analysis for ZC3H13 in various human cancers determined by the GEPIA database.** The OS and RFS plots of ZC3H13 in BRCA (**A**, **B**). The OS and RFS plots of ZC3H13 in KIRC (**C**, **D**). The OS and RFS plots of ZC3H13 in LIHC (**E**, **F**). The OS and RFS plots of ZC3H13 in LUAD (**G**, **H**). The OS and RFS plots of ZC3H13 in STAD (**I**, **J**).

Next, the prognostic impact of different expression levels of ZC3H13 on patients with BRCA, KIRC, LIHC, LUAD and STAD were analyzed. (Figure 2). The two prognostic indicators were overall survival (OS) and progression-free survival (RFS). If OS is used as an evaluation criterion, ZC3H13 can be used to assess the prognosis of patients with KIRC (P = 5.2E-07) and LIHC (P = 0.013). Again, with RFS assessment, only ZC3H13 is eligible for assessing prognosis in patients with LIHC (P = 0.046). No statistical significance was observed for ZC3H13 in predicting the prognosis in patients with other cancer types. In conclusion, by synthesizing differential expression and prognostic analysis results, ZC3H13 has met the screening criteria and can be used as a candidate factor to assess the prognosis of HCC patients.

### MiR-362-3p/miR-425-5p is an upstream miRNA of ZC3H13

**Figure 3:**
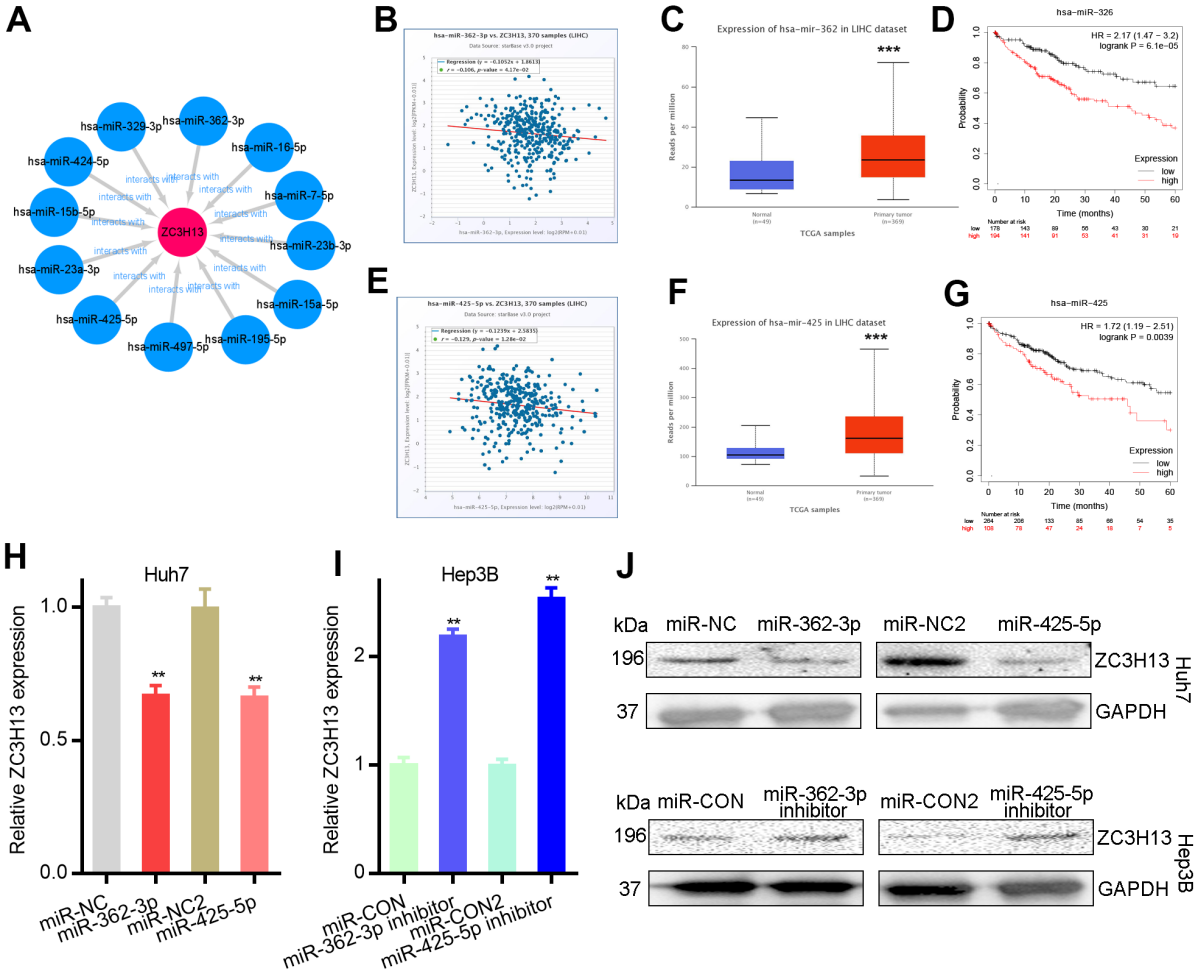
**Mir-362-3p/mir-425-5p downregulates ZC3H13 in liver cancer.** (**A**) The miRNA-ZC3H13 regulatory network established by Cytoscape software. (**B**) ZC3H13 is negatively correlated with miR-362-3p in LIHC analyzed by the starBase database. (**C**) Mir-362-3p is overexpressed in LIHC analyzed by UALCAN. (**D**) The prognostic value of miR-362-3p in HCC assessed by Kaplan–Meier plotter. (**E**) ZC3H13 is negatively correlated with miR-425-5p in LIHC analyzed by the starBase database. (**F**) Mir-425-5p is overexpressed in LIHC analyzed by UALCAN. (**G**) The prognostic value of miR-425-5p in HCC assessed by Kaplan–Meier plotter. (**H**, **I**) MiR-362-3p/miR-425-5p mimic and inhibitor results detected by qPCR. (**J**) MiR-362-3p/miR-425-5p mimic and inhibitor results detected by western blot. **P<0.01.

The ceRNA theory is used to describe the mechanism of regulating gene expression. Theoretically, ZC3H13 would be regulated by some ncRNAs; therefore, the upstream miRNAs to which ZC3H13 might be bound were first queried, and 12 miRNAs were identified (Table 1). Cytoscape software was used to establish the miRNA-ZC3H13 regulatory network (Figure 3A). Based on the mechanism by which miRNAs regulate target gene expression, a negative regulatory relationship should exist between miRNAs and ZC3H13; thus, correlation analysis was performed. ZC3H13 was inhibited by upstream miR-362-3p or miR-425-5p (Figure 3B, 3E). No negative correlation was found between ZC3H13 and the other nine predicted miRNAs. miRNAs are upregulated in liver cancer patients and function as oncogenes in terms of prognostic value. The above results are consistent with bioinformatics analysis, supporting the ceRNA hypothesis. Finally, based on the correlation analysis and prognosis analysis results of miR-362-3p/miR-425-5p and ZC3H13, the status of miR-362-3p/miR-425-5p as the upstream regulator of ZC3H13 was determined.

**Table 1:** The expression correlation between predicted miRNAs and ZC3H13 in HCC analyzed by starBase database.

**GeneName**	**miRNAname**	**R-value**	**P-value**
ZC3H13	hsa-miR-15a-5p	0.069	0.183
ZC3H13	hsa-miR-16-5p	0.173^a^	8.29E-04^a^
ZC3H13	hsa-miR-23a-3p	0.081	0.119
ZC3H13	hsa-miR-7-5p	0.026	0.616
ZC3H13	hsa-miR-15b-5p	0.021	0.691
ZC3H13	hsa-miR-23b-3p	0.036	0.492
ZC3H13	hsa-miR-195-5p	0.173^a^	8.35E-04^a^
ZC3H13	hsa-miR-424-5p	0.086	0.1
ZC3H13	hsa-miR-329-3p	0.006	0.905
ZC3H13	hsa-miR-497-5p	0.079	0.130
ZC3H13	hsa-miR-425-5p	-0.129^a^	0.0128^a*^
ZC3H13	hsa-miR-362-3p	-0.106^a^	0.0417^a*^

^a^These results are statistically significant.

*p value < 0.05; **p value < 0.01; ***p value < 0.001.

Huh7 and Hep3B cells were transfected with miR-362-3p/miR-425-5p, and the effect on ZC3H13 expression levels was tested. The downregulation of ZC3H13 expression was regulated by miR-362-3p/miR-425-5p mimics and qRT–PCR demonstrated that this reduction could be reversed by miR-362-3p/miR-425-5p inhibitors (Figure 3H, 3I). Furthermore, the protein levels of ZC3H13 were detected by western blotting. And the results were consistent with qRT–PCR results demonstrating that ZC3H13 protein levels were regulated by miR-362-3p/miR-425-5p mimics and inhibitors (Figure 3J). Strikingly, ZC3H13 was identified as a direct target of miR-362-3p/miR-425-5p, further corroborating the RNA-seq results (Figure 3).

### Determining the function of ZC3H13 in HCC

**Figure 4:**
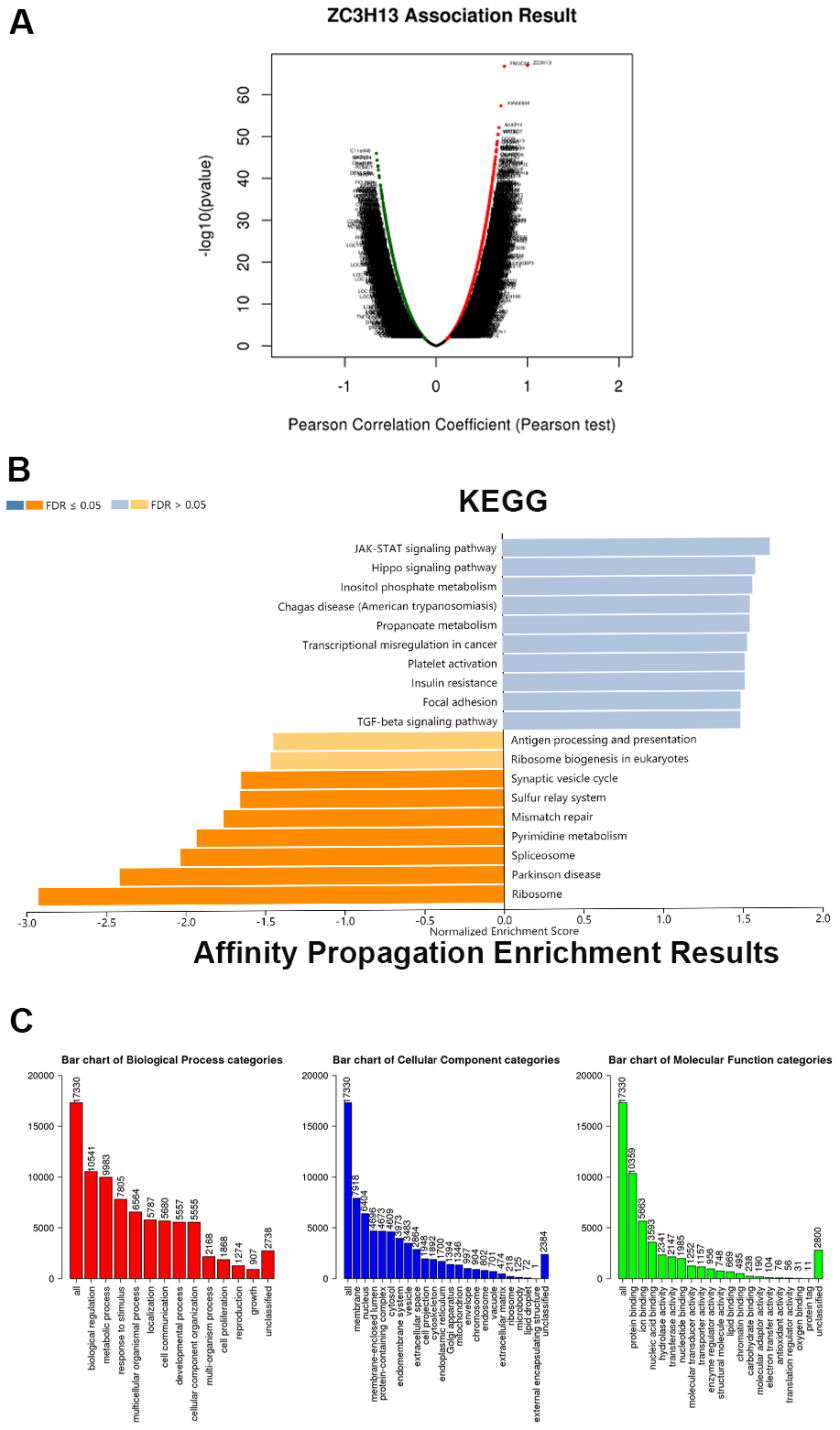
**ZC3H13 coexpression genes in LIHC (LinkedOmics).** (**A**) Global ZC3H13 highly correlated genes identified by the Pearson test in LIHC. Red and green dots represent positively and negatively significantly correlated genes with ZC3H13, respectively. (**B**) KEGG pathways of ZC3H13-correlated genes. (**C**) Gene Ontology of ZC3H13-correlated genes. KEGG: Kyoto Encyclopedia of Genes and Genomes; LIHC: Liver hepatocellular carcinoma.

To further explore the biological function and pathway analysis of the candidate factor ZC3H13 in HCC, TCGA sequencing data stored on LinkedOmics online tool were utilized. Red dots represent genes that are positively correlated with ZC3H13, and green dots represent negative correlations (Figure 4A, P < 0.05). The genes that were ranked in the top 50 positively and negatively associated with ZC3H13 were presented in a heat map in Supplementary Figure 1. ZC3H13 is involved in biological functions such as phosphoinositide metabolism, Chagas disease, propionate metabolism and transcriptional dysregulation in cancer. Kyoto Encyclopedia of Genes and Genomes (KEGG) analysis showed that these genes were mainly enriched in the JAK-STAT signaling pathway and Hippo signaling pathway.

### ZC3H13 positively correlates with immune cell infiltration in HCC

**Figure 5:**
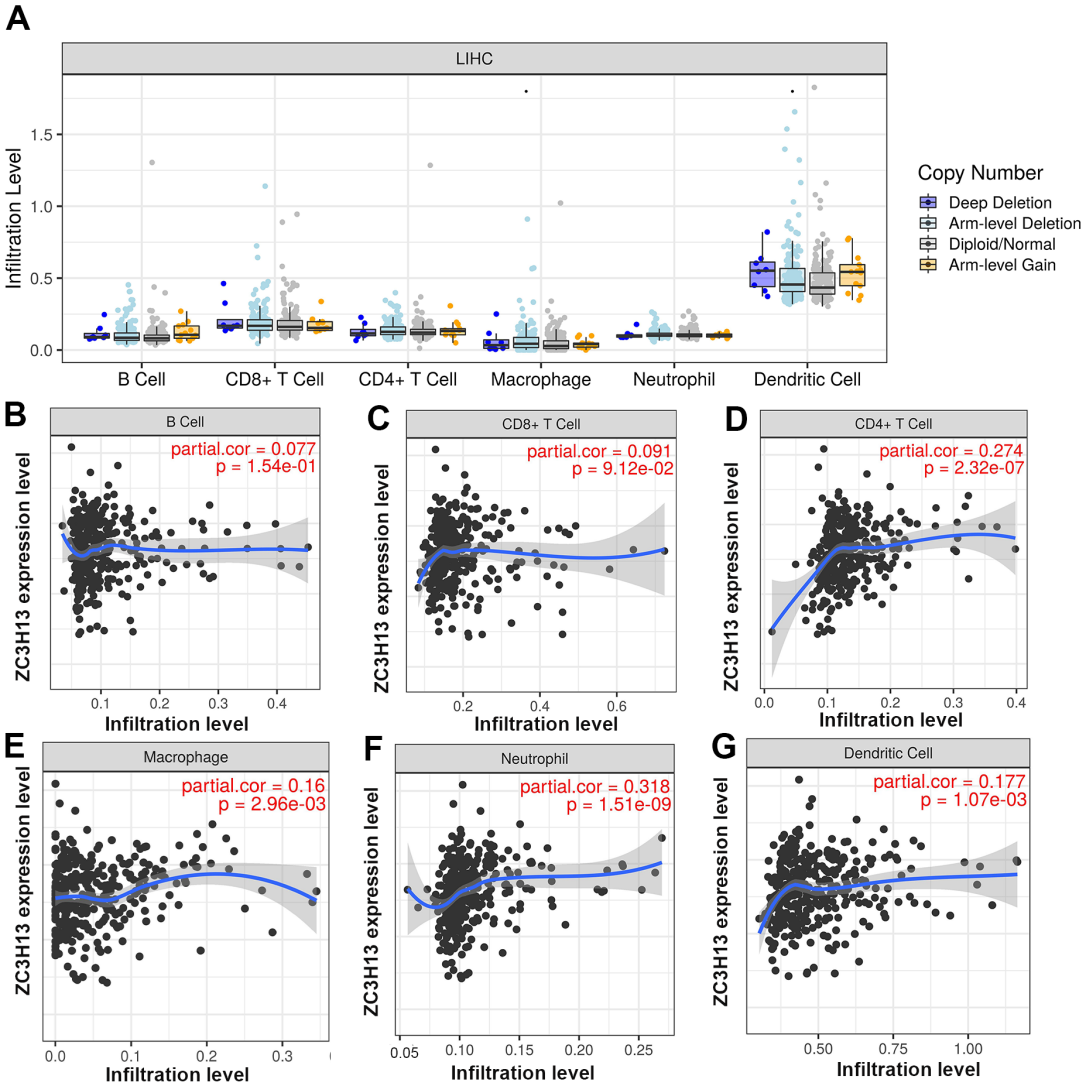
(**A**) The relationship of immune cell infiltration with ZC3H13 levels in HCC. (**B**–**G**) The infiltration level of various immune cells under different expression levels of ZC3H13 in HCC. The correlation of ZC3H13 expression level with B cell (**B**), CD8+T cell (**C**), CD4+T cell (**D**), macrophage (**E**), neutrophil (**F**), or dendritic cell (**G**) infiltration level in HCC.

Studies have identified zinc finger proteins in other diseases, and they are increasingly recognized as drug targets [14]. As a typical zinc finger protein, ZC3H13 has a finger domain. Tumor-infiltrating immune cells are represented by six types, namely B cells, CD8+ T cells, CD4+ T cells, macrophages, neutrophils and dendritic cells. Clarifying tumor-infiltrating immune cell types may help to improve the survival prediction of HCC patients increasing the therapeutic effect. Therefore, we investigated the correlation between ZC3H13 and tumor-infiltrating immune cells (Figure 5). The results revealed that the expression of ZC3H13 in HCC patients was significantly and positively correlated with four types of immune cell infiltration, including CD4+ T cells (P = 2.32E-07), macrophages (P = 2.96E-03), neutrophils (P = 1.51E-09) and dendritic cells (P = 1.07E-03). Among these immune cells, tumor-infiltrating neutrophils showed the strongest correlation.

### Correlation analysis of ZC3H13 and immune cell biomarkers in HCC

Continuing the correlation analysis in the previous section, this study analyzed the correlation between ZC3H13 and biomarkers expressed by immune cells in patients with hepatocellular carcinoma. The biomarkers expressed on immune cells are listed in Table 2. For example, CD4 is a CD4+ T cell biomarker. Correlation analysis showed that ZC3H13 was significantly positively correlated with CD4+ T cell biomarkers (R = 0.25, P = 1.60E-06), M1 macrophage biomarkers (R = 0.4, P = 1.20E-15), M2 macrophage biomarkers (R = 0.25, P = 1.10E-06), neutrophil biomarkers (R = 0.25, P = 7.50E-07), and dendritic cell biomarkers (R = 0.26, P = 4.00E-07). These findings prompted us to consider that ZC3H13 may lead to different prognostic outcomes of patients by influencing the immune cells infiltrating hepatocellular carcinoma patients.

**Table 2:** Correlation analysis between ZC3H13 and biomarkers of immune cells in HCC determined by GEPIA database.

**Immune cell**	**Biomarker**	**R value**	**p value**
B cell	CD19	-0.013	8.00E-01
	CD79A	0.1^a^	4.80E-02* ^a^
CD8+ T cell	CD8A	0.15^a^	4.20E-03** ^a^
	CD8B	0.033	5.20E-01
CD4+ T cell	CD4	0.25^a^	1.60E-06*** ^a^
M1 macrophage	NOS2	0.29^a^	1.70E-08*** ^a^
	IRF5	0.2^a^	7.50E-05*** ^a^
	PTGS2	0.4^a^	1.20E-15*** ^a^
M2 macrophage	CD163	0.07	1.80E-01
	VSIG4	0.18^a^	3.90E-04*** ^a^
	MS4A4A	0.25^a^	1.10E-06*** ^a^
Neutrophil	CEACAM8	0.078	1.30E-01
	ITGAM	0.24^a^	2.30E-06*** ^a^
	CCR7	0.25^a^	7.50E-07*** ^a^
Dendritic cell	HLA-DPB1	0.17^a^	1.10E-03** ^a^
	HLA -DQB1	-0.078	1.30E-01
	HLA-DRA	0.22^a^	1.60E-05*** ^a^
	HLA-DPA1	0.26^a^	4.00E-07*** ^a^
	CD1C	0.2^a^	1.20E-04*** ^a^
	NRP1	0.5^a^	1.40E-24*** ^a^
	ITGAX	0.28^a^	7.60E-08*** ^a^

^a^These results are statistically significant.

*p value < 0.05; **p value < 0.01; ***p value < 0.001.

### Relationship between ZC3H13 and immune checkpoints in HCC

PD1/PD-L1 and CTLA-4 are known to be key immune checkpoints, and they are responsible for tumor immune escape. We evaluated the correlation of ZC3H13 with these immune checkpoints in patients with hepatocellular carcinoma using bioinformatics. The analysis showed that the expression of ZC3H13 was significantly and positively correlated with PD-L1 in HCC (R = 0.437, P = 1.73E-17), which was adjusted by purity (Supplementary Figure 2). Consistent with the TIMER data analysis results, GEPIA database analysis found that ZC3H13 was positively correlated with PD-L1 (R = 0.33, P = 6.3E-11). In addition, no correlation was seen between ZC3H13 and PD1 and CTLA-4.

## DISCUSSION

The liver has a unique anatomical feature of dual blood supply, making liver cancer patients prone to recurrence or metastasis after surgery. This situation contributes to most advanced HCC patients being prone to a poor prognosis; thus, effective HCC diagnostic and prognostic biomarkers must be identified [7, 15].

Presently, domestic and foreign researchers have proposed the following views: ZC3H13 has the potential to be a prognostic indicator or therapeutic target for hepatocellular carcinoma [13]. However, the role of ZC3H13 in hepatocellular carcinoma remains controversial. Liu Jun et al. evaluated the transcriptome data and corresponding clinical data of m6A methylation-related genes (including 15 genes) from the TCGA database and found that ZC3H13 was upregulated in high-risk tumor patients and that ZC3H13 was associated with survival and prognosis, making it a potential predictor for patients with hepatocellular carcinoma [16]. However, Wu Xiaomin et al. also used The Cancer Genome Atlas (TCGA) database for bioinformatics analysis, but they proposed the opposite point of view: ZC3H13 was overexpressed in hepatocellular carcinoma patients, and they used m6A regulators such as YTHDF2, YTHDF1, METTLl3, KIAA1429, and ZC3H13 as prognostic features, which could successfully distinguish high-risk patients with HCC [17]. Although the above articles reported the expression of ZC3H13 in HCC, they lacked the regulatory mechanism of ZC3H13 expression.

In the present study, also based on the TCGA dataset and bioinformatics analysis, ZC3H13 was expressed at low levels in HCC patients. ZC3H13 is a “protective factor” for patients with hepatocellular carcinoma, and patients with low ZC3H13 expression have a poor prognosis. To further elucidate the regulatory mechanism of ZC3H13, we explored upstream regulatory factors.

The ceRNA mechanism involves three levels of mRNA, miRNA and lncRNA and regulates gene expression [18, 19]. We predict upstream miRNAs that can regulate ZC3H13 through the starBase database. Finally, 12 miRNAs were obtained from the screening. Most miRNAs act as oncogene miRNAs in HCC. Considering the above analysis, after continuing to perform correlation analysis, expression analysis and survival analysis on this basis, we identified miR-362-3p/miR-425-5p as the ZC3H13 upstream oncogene miRNA with the most potential. Previous studies have reported that miR-362-3p/miR-425-5p exerts oncogenic effects by regulating HCC proliferation and migration. For example, miR-425-5p achieved its role in promoting the malignant progression of HCC by regulating RNF11 and was seen as a molecular target to predict the outcome of HCC patients [20]. Upregulation of miR-362-3p, which targets and regulates Tob2, thereby promoting the proliferation of hepatocellular carcinoma cells [21]. Li, Z et al. found that inhibition of Rab23 expression by overexpression of miR-362-3p inhibited the growth and proliferation of Hep3B cells *in vivo* [22].

The mechanism of ZC3H13 downregulation in HCC was explained by our results. MiR-362-3p/miR-425-5p bound to the ZC3H13 3’ UTR (Supplementary Figure 3), validating database findings that miR-362-3p/miR-425-5p targets and regulates ZC3H13 expression. Additionally, miR-362-3p/miR-425-5p expression was inversely associated with ZC3H13 expression in HCC tissues.

Many studies have confirmed that the therapeutic efficacy and prognosis of cancer patients are closely related to tumor immune cell infiltration. Our work showed that ZC3H13 is positively associated with the following four types of immune cell infiltration in HCC: CD4+ T cells, macrophages, neutrophils and dendritic cells. More precisely, ZC3H13 was also positively correlated with the above biomarkers expressed on immune cells. These findings suggested that tumor immune infiltration may partially explain the different ZC3H13 expression levels affecting the prognosis of patients with hepatocellular carcinoma.

## CONCLUSIONS

Considering the controversy over ZC3H13 expression in HCC patients, this study showed that ZC3H13 is downregulated in HCC and significantly associated with a poor prognosis in HCC. To elucidate the mechanism of ZC3H13 downregulation in hepatocellular carcinoma, the miR-362-3p/miR-425-5p-ZC3H13 axis was further evaluated. In addition, ZC3H13 may affect the prognosis of patients with hepatocellular carcinoma by increasing four tumor immune cell (CD4+ T cells, macrophages, neutrophils and dendritic cells) infiltration and thus affecting the prognosis of patients with hepatocellular carcinoma. However, more basic trials and large clinical trials will make these findings even more convincing.

## MATERIALS AND METHODS

### Predicted upstream regulators of ZC3H13

StarBase (http://starbase.sysu.edu.cn/) searches were performed for microRNA targets using high-throughput CLIP-Seq experimental data and degradome experimental data, providing various visual interfaces to explore microRNA targets. The miRNA-mRNA module was used to analyze upstream miRNAs [23]. StarBase database involves PITA, RNA22, miRmap, microT, miRanda, PicTar, and TargetScan target gene prediction programs. Only target miRNAs validated by more than two prediction programs were considered as miRNAs meeting the screening criteria. The miRNAs screened above were considered candidate miRNAs of ZC3H13. And starBase was used to analyze the correlation between miR-362-3p/miR-425-5p and ZC3H13 in liver cancer patients. Comparison of miR-362-3p/miR-425-5p expression differences between hepatocellular carcinoma patients and normal was also done through this website.

### Oncomine analysis

The Oncomine database is a large oncology gene microarray database. Because it covers 65 gene microarray datasets, large-scale gene expression data are recognized and used by everyone. This study used it to analyze gene expression differences. It can also have functional modules such as identifying abnormal values and predicting co-expressed genes [24]. In this study, we analyzed the differences in expression of m6A-related genes in HCC patients and healthy individuals based on mRNA levels using the Oncomine database. Analysis was based on the following criteria: fold change = 2 and P value = 0.01.

### GEPIA database analysis

GEPIA (http://gepia.cancer-pku.cn/) integrates TCGA cancer data with GTEx normal tissue data. By revealing driver genes, alleles, differential expression or oncogenic factors, it can provide key clues for our analysis of novel cancer targets and markers. Therefore, it is often used to address differential gene expression in cancer biology [25]. The expression of ZC3H13 in different types of human tumors was detected by GEPIA. In addition, the relationship of ZC3H13 with immune cell surface markers and immune checkpoints in patients with hepatocellular carcinoma was evaluated. The selection criteria were statistically significant with R > 0.1 and a p value < 0.05.

### Kaplan–Meier plotter analysis

The Kaplan–Meier plotter (http://kmplot.com) database is processed by a PostgreSQL server that integrates both gene expression and clinical data [26]. Gene expression data and recurrence-free and overall survival information were downloaded from GEO, EGA, and TCGA. To analyze the prognostic value of ZC3H13 and upstream miRNAs, samples of hepatocellular carcinoma patients were divided into two groups with high and low expression. The two cohorts were compared by Kaplan-Meier survival plots, and hazard ratios and log-rank P values were calculated using 95% confidence intervals. Survival time was selected as 60 months.

### TIMER database analysis

The TIMER database (https://cistrome.shinyapps.io/timer/) stores a comprehensive resource of immune infiltrations of different cancer types. It is one of the common databases used to analyze tumor immune infiltrates. This study involved six immune cells containing B cells, CD4+ T cells, CD8+ T cells, neutrophils, macrophages and dendrites [27]. We analyzed the correlation between ZC3H13 and immune cell infiltration levels or immune checkpoint expression levels in liver cancer patients. A p value < 0.05 was considered statistically significant.

### Gene set enrichment analysis

LinkedOmics (http://www.linkedomics.org) has over a billion data points. Specifically, it contains the TCGA dataset covering 32 cancer types and stores multi-omic and clinical data on more than 10,000 patients [28]. The “LinkInterpreter” module can translate big data about ZC3H13 into biological functions and enumerate the most likely signaling pathways involved through its inherent pathway analysis and network analysis. It was used for Gene Ontology and Kyoto Encyclopedia of Genes and Genomes (KEGG) pathway analysis by Gene Set Enrichment Analysis (GSEA). In the “LinkFinder” module of LinkedOmics, we performed a statistical analysis of ZC3H13 co-expression using the Pearson test, and the results are presented in the form of volcano plots. The rank criterion was a false discovery rate (FDR) < 0.05, and there were 500 simulations.

### UALCAN database analysis

UALCAN (http://ualcan.path.uab.edu/index.html) is also based on the TCGA database of relevant cancer data. It is often used for online analysis and mining of cancer data [29]. It can perform commands such as expression profiling, survival analysis, correlation analysis, methylation levels and even target gene prediction. Gene expression of miR-362-3p/miR-425-5p in the LIHC TCGA dataset was explored using UALCAN. P < 0.05 indicated statistical significance.

### Cell culture, cell transfection, RNA isolation and quantitative real-time PCR (qPCR) analysis

The HCC cell lines used in this study were purchased from the American Type Culture Collection and included Hep3B and Huh7 cells. All cancer cells survived in an environment based on high glucose Dulbecco’s modified Eagle’s medium (DMEM; Thermo Scientific) supplemented with 10% fetal bovine serum (FBS, Gibco), 0.1 mmol/L MEM nonessential amino acids (NEAA; Invitrogen) and 1% L-glutamine (Invitrogen). All the cell lines were cultured under growth conditions of 5% CO_2_ in an incubator at 37° C and 100% humidity. The cells were transfected at a density of 2 × 10^6^/ml with the indicated miRNA mimics or inhibitors using Lipofectamine® 3000 (Invitrogen; Thermo Fisher Scientific, Inc.) as indicated by the manufacturer. After 48 hours, the cells were used for subsequent experiments.

Total RNA was extracted from HCC cells using TRIzol® Reagent (Invitrogen; Thermo Fisher Scientific, Inc.) following the manufacturer’s instructions. First, total RNA (1 μg) was reverse transcribed into cDNA using the PrimeScript RT kit with gDNA Eraser (Takara Biotechnology Co., Ltd.) (Experimental conditions: 37° C for 15 minutes and 85° C for 5 seconds). Subsequently, qPCR was performed using TB Green Fast qPCR mix (Takara Biotechnology Co., Ltd.). The following primers were used for qPCR: ZC3H13 forward, 5’- AAAGGAGGGTTTCACCAGAAGTG-3’ and reverse, 5’- CGCTTCGGAGATTTGCTAGAC-3’; and GAPDH forward, 5’- GAAATCCCATCACCATCTTCCAGG-3’ and reverse, 5’- GAGCCCCAGCCTTCTCCATG- 3’. qPCR was performed under the following thermal cycling conditions: initial denaturation at 95° C for 2 min, followed by 40 cycles of 95° C for 20 s, 58° C for 20 s, and 72° C for 20 s. The mRNA expression levels were quantified using the 2-ΔΔCt method and normalized to the internal reference gene GAPDH.

### Western blotting

Cells were collected and washed twice with PBS. The cells were then lysed with RIPA lysis buffer containing protein inhibitors on ice for 30 minutes. The cell lysate was then centrifuged at 13 000 rpm for 30 minutes. Supernatants containing protein were collected, and the protein was quantitated using a Pierce BCA Protein Assay Kit (Thermo Fisher Scientific, Waltham, MA). SDS-PAGE involves the principle of separating proteins by electrophoresis according to their size. After electrophoresis, the proteins are transferred to polyvinylidene difluoride (PVDF) membranes. The membranes were blocked with 5% skim milk and shaken at room temperature for 30 minutes. The primary antibody targeting the protein of interest was incubated with the membrane overnight at 4° C with shaking. The next day, an HRP-labeled secondary antibody was used to detect the protein of interest. Finally, the bands were visualized using Pierce ECL (Thermo Fisher Scientific, Waltham, MA). All of the images were captured using the Molecular Imager ChemiDoc XRS+ (Bio–Rad, Hercules, CA).

### Statistical analysis

Statistical analyses were automatically calculated from the aforementioned online databases and were divided into the following four aspects: (1) Comparison of mRNA expression using Student’s t test; (2) Correlation of gene expression using Spearman’s correlation to assess or infiltrate immune cells and Pearson’s correlation method; (3) Fisher’s exact test to measure gene enrichment; (4) a p value < 0.05 or a log rank p value < 0.05 was considered statistically significant.

### Data availability

The datasets presented in this study can be found in publicly available databases. The names of the databases and their websites have been marked in the article.

## Supplementary Material

Supplementary Figures
