# Supplementary material for: Downregulation of ZC3H13 by miR-362-3p/miR-425-5p is associated with a poor prognosis and adverse outcomes in hepatocellular carcinoma
Source: Aging (Albany NY). 2022 Mar 12;14(5):2304–19. doi: 10.18632/aging.203939 (PMC8954979; doi:10.18632/aging.203939)
Supplement: Supplementary Figures [file aging-14-203939-s001.pdf]

SUPPLEMENTARY FIGURES

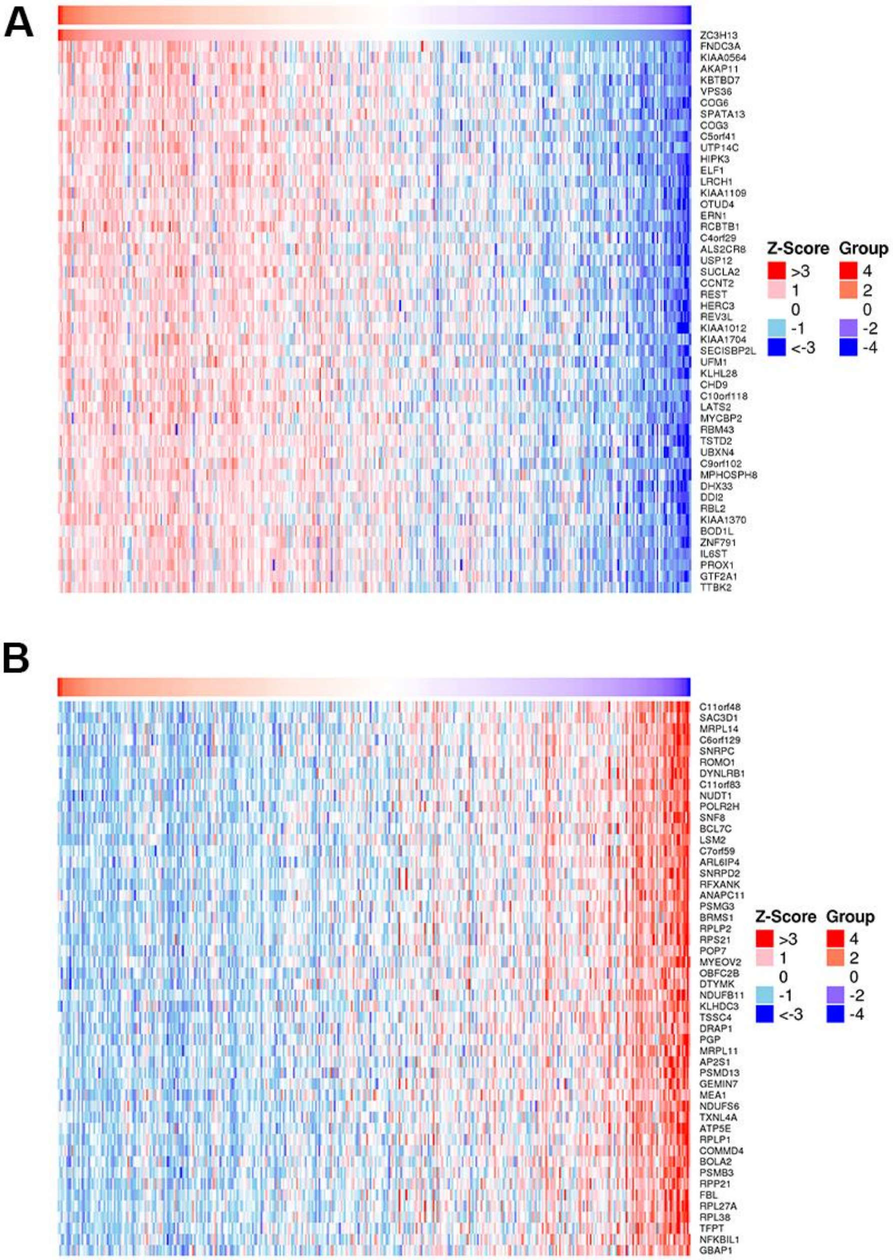

Supplementary Figure 1. (**A**, **B**) Heatmaps showing the top 50 genes positively and negatively correlated with ZC3H13 in LIHC.

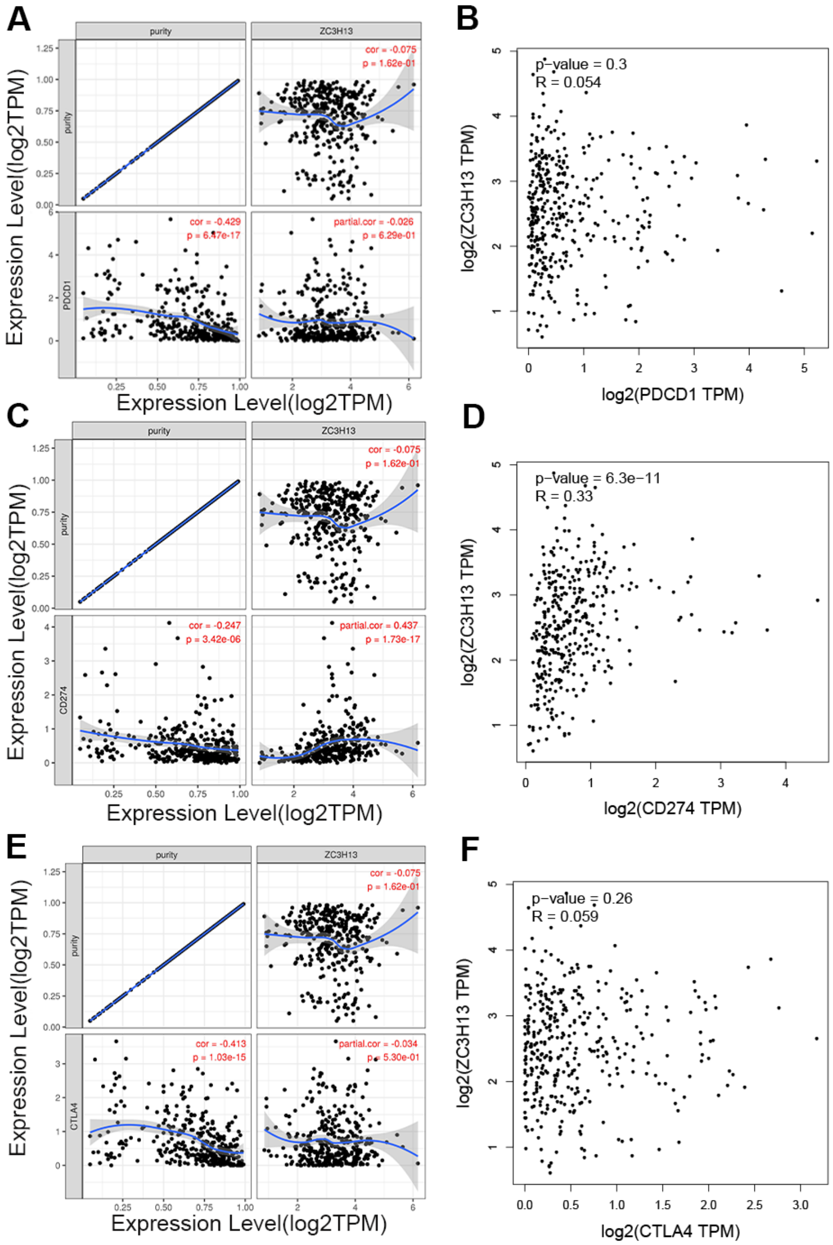

Supplementary Figure 2. Correlation of ZC3H13 expression with PD-1, PD-L1, and CTLA-4 expression in HCC. (**A**) Spearman correlation of ZC3H13 with the expression of PD-1 in HCC adjusted by purity using TIMER. (**B**) The expression correlation of ZC3H13 with PD-1 in HCC determined by the GEPIA database. (**C**) Spearman correlation of ZC3H13 with the expression of PD-L1 in HCC adjusted by purity using TIMER. (**D**) The expression correlation of ZC3H13 with PD-L1 in HCC determined by the GEPIA database. (**E**) Spearman correlation of ZC3H13 with the expression of CTLA-4 in HCC adjusted by purity using TIMER. (**F**) The expression correlation of ZC3H13 with CTLA-4 in HCC determined by the GEPIA database.

Binding Site of hsa-miR-362-3p on ZC3H13:

Show 10 entries

<table>
<tr><th>BindingSite</th><th>Class</th><th>Alignment</th></tr>
<tr><td>chr13:46537300-46537305[-]</td><td>6mer</td><td>Target: 5' cuAUUACUU-AAUCUGUGUGUu 3'
| | ||| ||| ||||||
miRNA : 3' acUUAGGAACUUAUCCACACAa 5'</td></tr>
</table>

Binding Site of hsa-miR-362-3p on ZC3H13:

Show 10 entries

<table>
<tr><th>BindingSite</th><th>Class</th><th>Alignment</th></tr>
<tr><td>chr13:46537403-46537410[-]</td><td>8mer</td><td>Target: 5' caucuacccaAAUGCAGGUGUGUa 3'
||| ||||||||
miRNA : 3' acuuaggaacUUA--UCCACACAa 5'</td></tr>
</table>

Binding Site of hsa-miR-425-5p on ZC3H13:

Show 10 entries

<table>
<tr><th>BindingSite</th><th>Class</th><th>Alignment</th></tr>
<tr><td>chr13:46537291-46537297[-]</td><td>7mer-m8</td><td>Target: 5' uuAAUCUGUGUGUUAGUGUCAUc 3'
||: | ||| | |||||||
miRNA : 3' agUUGCCCUCACUAGCACAGUAa 5'</td></tr>
</table>

Supplementary Figure 3. The binding site of miR-362/miR-425 to ZC3H13 was determined by the starBase database.
